# The molecular mechanisms of defensive‐grade organic acid biosynthesis in ground beetles

**DOI:** 10.1111/imb.12984

**Published:** 2025-02-10

**Authors:** Adam M. Rork, Sihang Xu, Athula Attygalle, Tanya Renner

**Affiliations:** ^1^ Department of Entomology Purdue University West Lafayette Indiana USA; ^2^ Department of Entomology The Pennsylvania State University University Park Pennsylvania USA; ^3^ Department of Chemistry and Chemical Biology Stevens Institute of Technology Hoboken New Jersey USA; ^4^ Department of Biological and Chemical Sciences New York Institute of Technology New York New York USA

**Keywords:** Carabidae, evolution, exocrine, folate cycle, valine catabolism

## Abstract

Insects are known to synthesise and secrete hundreds of unique defensive chemicals, including caustic acids, pungent phenolics and citrusy terpenes. Despite efforts to characterise the defensive chemistry of ground beetles (Coleoptera: Carabidae), our knowledge of semiochemical evolution within the family and how these compounds are biosynthesised remains limited. Few studies have demonstrated the likely biosynthetic precursors of select compounds in certain taxa and only one has demonstrated which genes may be involved in the biosynthesis of formic acid. Here, we characterise the defensive chemistry and generate defensive gland transcriptomes for ground beetle species representing two defensive chemical classes: the formic acid producer *Platynus angustatus* and the methacrylic acid producer *Pterostichus moestus*. Through comparative transcriptome analyses, we demonstrate that co‐option of distinct primary metabolic pathways may be involved in formic acid and methacrylic acid biosynthesis in the defensive glands of these taxa. These results expand our knowledge of ground beetle defensive chemistry and provide additional evidence that co‐option of existing primary metabolic pathways plays a major role in the evolution of ground beetle chemical defence.

## Graphical abstract

- Formic acid and methacrylic acid are the primary defensive chemicals in the pygidial gland secretions of *Platynus angustatus* and *Pterostichus moestus*, respectively.
- Comparative transcriptomics suggests the folate cycle and valine catabolism may be involved in the biosynthesis of formic acid and methacrylic acid, respectively.
- A probable serine hydrolase has been identified as a potential candidate gene involved in the biosynthesis of methacrylic acid from methacrylyl‐CoA.

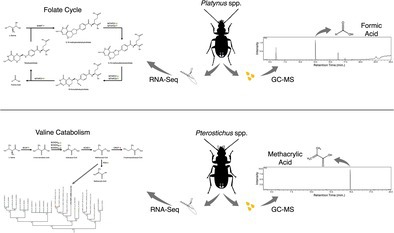

## INTRODUCTION

Arthropods are well known for not only their taxonomic diversity, but their biochemical diversity (Roth & Eisner, 1962). For many species, biochemicals are the primary means of communicating ecologically relevant information with conspecifics and heterospecifics alike. Eusocial insects, such as ants, emit trail pheromones to inform nestmates about the location of resources (Scarano et al., 2024). Male Lepidoptera emit sex pheromones from coremata to advertise their location to potential mates as well as repel competitors (Mullegama & Hiller, 2023). Bark beetles (Scolytinae) are well known for emitting aggregation pheromones that attract swarms of conspecifics to trees for oviposition, resulting in vast quantities of larvae feeding upon the tree and overwhelming induced defences (Byers et al., 2013). Millipedes secrete toxic quinones, cyanide and benzaldehyde in response to perturbation by organisms ignoring their aposematism (Rodriguez et al., 2018). These semiochemicals have been of interest to the scientific community for decades given their potential to explain patterns of insect evolution and behaviour. Amongst the most well studied are insect defensive compounds, which include small organic molecules, venoms and complex polymers (Attygalle et al., 1993; Eisner et al., 1961; Laxme et al., 2019; Roth & Eisner, 1962). The former comprise arguably the largest area of inquiry and include a wide variety of metabolites such as carboxylic acids, quinones, phenolics, terpenes and sulfides (Moore & Wallbank, 1968; Roth & Eisner, 1962). Indeed, hundreds if not thousands of small organic defensive compounds have been described across Arthropoda, mostly from exocrine secretions. Despite our breadth of knowledge regarding the diversity of insect defensive compounds, our knowledge of their biosynthesis and evolution is wanting. Efforts have been made in recent years to fill this gap in knowledge, perhaps most notably in one of the phylum's most diverse lineages, Adephaga (Coleoptera).

**FIGURE 1:**
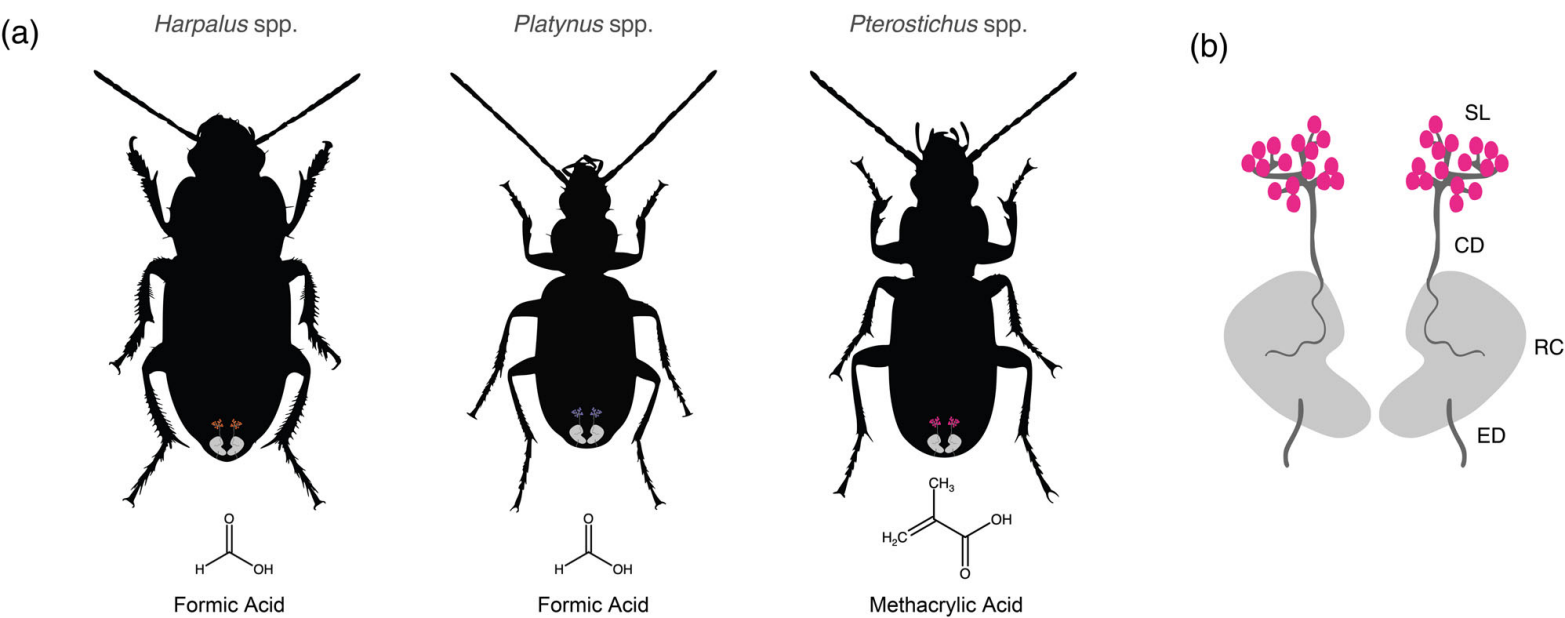
(a, b) Silhouette representations of *Harpalus* sp., *Platynus* sp. and *Pterostichus* sp. with graphical representations of the pygidial glands shown at approximate locations in the abdomen (a), along with the primary defensive compound they secrete (formic or methacrylic acid). An enlarged representation of the pygidial glands is displayed in (b), noting the generalised organisation consisting of the secretory lobes (SL), collecting duct (CD), reservoir chamber (RC) and efferent duct (ED). Neither glands nor species are to scale. Silhouettes are based on photographs of *Harpalus smaragdinus*, *Platynus livens* and *Pterostichus macer* taken by Udo Schmidt (original photos on Flickr under a CC BY‐SA 2.0 licence).

A synapomorphy of Adephaga is the unique pair of defensive glands situated in the abdomen, aptly known as pygidial glands (Figure 1a) (Forsyth, 1968; Forsyth, 1972). These glands are often composed of four gross morphological units, although additional features have evolved in some lineages. These include the secretory lobes, the collecting ducts, the reservoirs, and the efferent ducts (Figure 1b). Defensive chemical precursors are transported into secretory lobe tissue, the primary site of defensive chemical biosynthesis. Defensive compounds are then secreted from the secretory lobes into the chemically resistant, resilin‐rich collecting ducts to the reservoirs for storage (Forsyth, 1972; Muzzi et al., 2019; Rork et al., 2019). When a beetle is perturbed, the reservoirs contract, propelling defensive compounds through the efferent ducts which open to the tip of the abdomen. In certain taxa, additional accessory glands have been found articulated to the efferent duct (Forsyth, 1972). The efferent ducts of bombardier beetles (Brachininae and Paussinae) have evolved into sclerotised reaction chambers within which their characteristically exothermic conversion of p‐hydroquinones and hydrogen peroxide to p‐benzoquinones and water occurs (Aneshansley et al., 1969). In other taxa, the reservoir is divided into two lobes rather than the one typical of most taxa (Will et al., 2000).

Adephagan defensive chemistry is exceptionally diverse, with several hundred chemicals described and likely more to be discovered (Rork & Renner, 2018). These compounds can be categorised into broad chemical classes, including carboxylic acids, quinones, terpenes, sulphides, aromatic acids and hydrocarbons. Of these, the most ubiquitous appear to be the carboxylic acids, namely, methacrylic acid and formic acid (Kanehisa & Murase, 1977; Will et al., 2000). Methacrylic acid is present in several adephagan subfamilies, including the Carabinae, Harpalinae, Scaratinae and Trechinae (Kanehisa & Murase, 1977; Lečić et al., 2014; Moore & Wallbank, 1968). Formic acid has only been detected in the Harpalinae and Trechinae but is especially widespread in the former (Kanehisa & Murase, 1977, Will et al., 2000). Also widespread are quinones, which while not as ubiquitous as formic or methacrylic acid, can be found in at least three subfamilies: the Brachininae, the Harpalinae and the Paussinae (Kanehisa & Murase, 1977; Moore & Wallbank, 1968; Schildknecht et al., 1968). The Harpalinae biosynthesise benzoquinones directly in the secretory lobes whereas the Brachininae and Paussinae oxidise hydroquinones to benzoquinones in their reaction chambers. While often not discussed as primary defensive chemicals, aliphatic hydrocarbons, ketones, aldehydes, esters and alcohols can be found in the pygidial gland secretions of most subfamilies (Will et al., 2000).

Given the most current phylogenetic hypotheses for the family Carabidae, this pattern of defensive chemical presence across the family suggests many compounds have evolved several times independently (Vasilikopoulos et al., 2021). Indeed, quinones are likely to have evolved at least three times, formic acid at least twice, terpenes twice, etc. (Attygalle et al., 2009; Moore & Brown, 1979). Even the highly specialised bombardier beetle phenotype, which entails the biosynthesis of concentrated (>20%) hydrogen peroxide, has evolved twice in the family (Di Giulio et al., 2015; Muzzi et al., 2019; Vasilikopoulos et al., 2021). This raises an interesting question about the nature of defensive chemical evolution in the Carabidae: where independent lineages have evolved to secrete identical defensive chemicals, do they biosynthesise these compounds using identical or distinct biochemical pathways? For example, transcriptomic evidence demonstrates that the formic acid producer *Harpalus pensylvanicus* may biosynthesise its primary defensive compound via the folate cycle of C1 metabolism (Rork et al., 2021). However, there are several other mechanisms by which formic acid could be biosynthesised in Carabidae, including the kynurenine pathway, the methionine salvage cycle and alpha‐oxidation of fatty acids (Badawy, 2017; Brosnan & Brosnan, 2016; Meiser et al., 2016). Perhaps the folate cycle was not only co‐opted by *H. pensylvanicus*, but all formic acid‐producing carabids. Alternatively, the folate cycle may have been co‐opted in some taxa, the kynurenine pathway in others, etc. It may also be that some lineages biosynthesise formic acid through novel pathways. Unfortunately, aside from *H. pensylvanicus* and a few other taxa, we know very little about how carabid defensive chemicals are biosynthesised (Adachi et al., 1985; Attygalle et al., 1991; Attygalle et al., 2006; Attygalle et al., 2020; Rork et al., 2021).

Here, we aim to identify candidate genes and pathways underlying formic acid and methacrylic acid biosynthesis for two taxa belonging to the subfamily Harpalinae. We hypothesise that the formic acid producer *Platynus angustatus* (Platynini) upregulates genes involved in the folate cycle of C1 metabolism within their secretory lobes, suggesting a role in the biosynthesis of defensive‐grade formic acid as in the case of *Harpalus pensylvanicus* (Rork et al., 2021). Furthermore, we hypothesise that *Pterostichus moestus* (Pterostichini) biosynthesises methacrylic acid from L‐valine and thus would upregulate genes involved in the valine catabolic pathway. This is based on previous work which has shown that the species *Carabus yaconinus* (Carabinae) and *Scarites subterraneus* (Scaratinae), biosynthesise methacrylic acid from L‐valine (Adachi et al., 1985; Attygalle et al., 1991). The valine catabolic pathway does not typically lead to the biosynthesis of methacrylic acid but does generate the immediate fatty acyl‐CoA precursor, methacrylyl‐CoA, a probable intermediate in the acid's biosynthesis. We also aimed to identify genes involved in defensive carboxylic acid transport in these species' secretory lobes. Without efficient export of these compounds from gland cells into collecting duct lumens, not only would these species have no chemical stores to defend themselves with, but the glands themselves would be subject to immense physiological stress due to the buildup of cytotoxic chemicals.

## RESULTS

### GC–MS analysis of pygidial gland defensive chemicals

**FIGURE 2:**
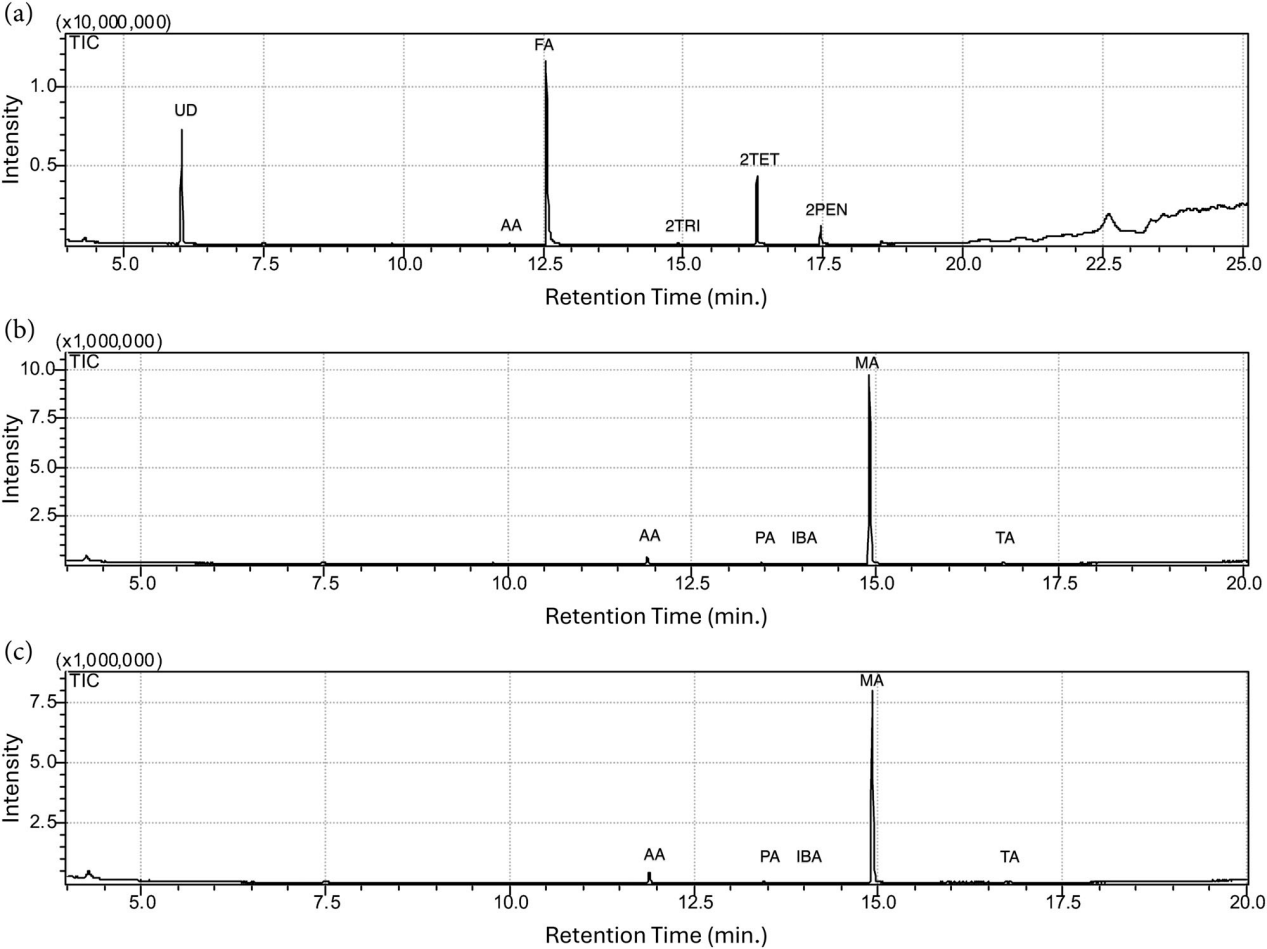
(a, b) Gas chromatographs of the pygidial gland secretions of *Platynus angustatus* (a) and *Pterostichus moestus* (b: Female, c: Male). Peaks corresponding to organic acids are as follows: AA) Acetic acid, FA) Formic acid, PA) Propanoic acid, IBA) Isobutyric acid, MA) Methacrylic acid, TA) Tiglic acid. Peaks corresponding to hydrocarbons and ketones are as follows: UD) Undecane, 2TRI) 2‐tridecanone, 2TET) 2‐tetradecanone, 2PEN) 2‐pentadecanone. Retention time (in minutes) is displayed along the x‐axis and peak intensity is displayed along the y‐axis.

The pygidial glands of *Platynus angustatus* contain formic acid, acetic acid, undecane, 2‐tridecanone, 2‐tetradecanone and 2‐pentadecanone (Figure 2a). To our knowledge, this is the fourth known instance of formic acid being produced by members of the genus *Platynus*, the second instance of 2‐tridecanone detection and the second of undecane detection (Kanehisa & Kawazu, 1985). The remaining three compounds had not been detected in the pygidial gland defensive secretions of *Platynus* spp. to date (Kanehisa & Kawazu, 1985; Kanehisa & Murase, 1977; Schildknecht, 1970). In the pygidial gland secretions of *Pterostichus moestus* a total of five compounds were detected: acetic acid, propanoic acid, isobutyric acid, methacrylic acid and tiglic acid (Figure 2b,c). This is the 24th known instance of methacrylic and tiglic acids in *Pterostichus* spp., the second instance of acetic acid and 2‐methylpropanoic acid detection and the first of propanoic acid detection (Kanehisa & Kawazu, 1982; Kanehisa & Murase, 1977; Schildknecht, 1970; Will et al., 2000).

### RNA‐Seq statistics

We discarded little data in terms of total reads across all samples (1.9%–2.6%). For *Harpalus pensylvanicus*, we removed approximately 5.7% of all bases. Due to the higher adapter content for *Platynus angustatus* (46.1%) and *Pterostichus moestus* (47.7%), we removed 13.2% and 15.1% of bases for those species respectively. A full list of summary statistics for the raw and trimmed RNA‐Seq data can be found in Supplementary Table S1.

### Transcriptome assembly statistics

All transcriptomes were almost entirely complete according to BUSCO (97.4%–98.5%). The majority of BUSCOs were duplicated (64.1% to 92.8%) due to the assembly of multiple isoforms per gene (Simāo et al., 2015).

*Pterostichus moestus* had the smallest number of assembled transcripts (105,544) and the shortest total assembly length (123 Mb). *Harpalus pensylvanicus* had the largest assembly (163,238 transcripts, 167 Mb assembly size). GC content was similar for all species, ranging from 36.3% to 38.4%. E90N50 values ranged from 1649 bp to 1750 bp.

Between 44.8% and 47.7% of all transcripts were predicted to contain at least one coding sequence. Across species, between 32.8% and 35.8% of transcripts received one or more blastx hits, between 63.9% and 66.1% of all predicted proteins received one or more blastp hits and between 59.7% and 60.4% of all predicted proteins received one or more hmmscan hits.

Per species, the average pseudoalignment rate across all samples was between 68.0% and 81.9%, the lowest being in *Harpalus pensylvanicus* and the highest in *Pterostichus moestus*. Differential gene expression analyses revealed between 469 and 1010 genes to be significantly upregulated in the secretory lobes of all species with *P. angustatus* having the fewest upregulated genes and *H. pensylvanicus* having the most. A full list of summary statistics for the transcriptome assembly and downstream analyses can be found in Supplementary Tables S2–S5.

### Candidate genes and pathways involved in formate biosynthesis

**FIGURE 3:**
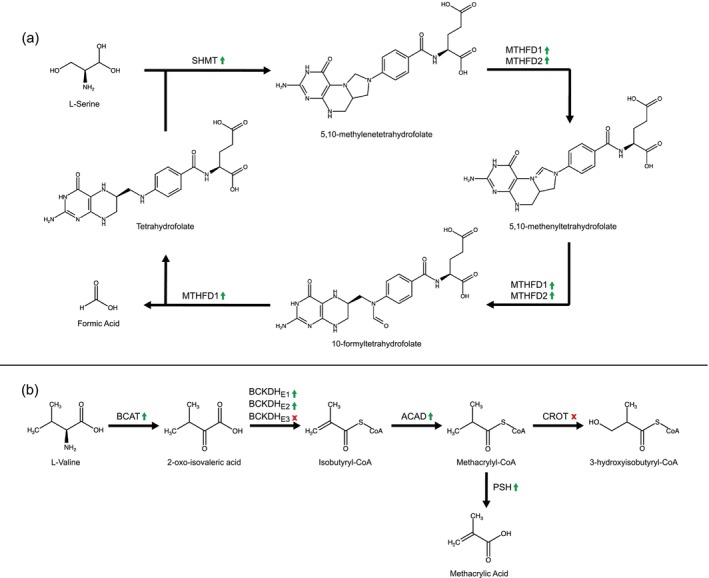
(a, b) Simplified representations of the folate cycle of one‐carbon metabolism (a) and the valine catabolic pathway (b). Compound names are shown below structures and gene/enzyme abbreviations above bolded black arrows. In (b), green arrows pointed upward to the right of gene/enzyme abbreviations indicate significant upregulation (logFC >4, FDR <0.05) in the secretory lobes of *H. pensylvanicus* and *P. angustatus*. In (b), the green arrows have the same meaning, but indicate upregulation in the secretory lobes of *P. moestus*. A red x to the right of the gene/enzyme abbreviations indicate that it is not significantly upregulated in *P. moestus* secretory lobes.

**FIGURE 4:**
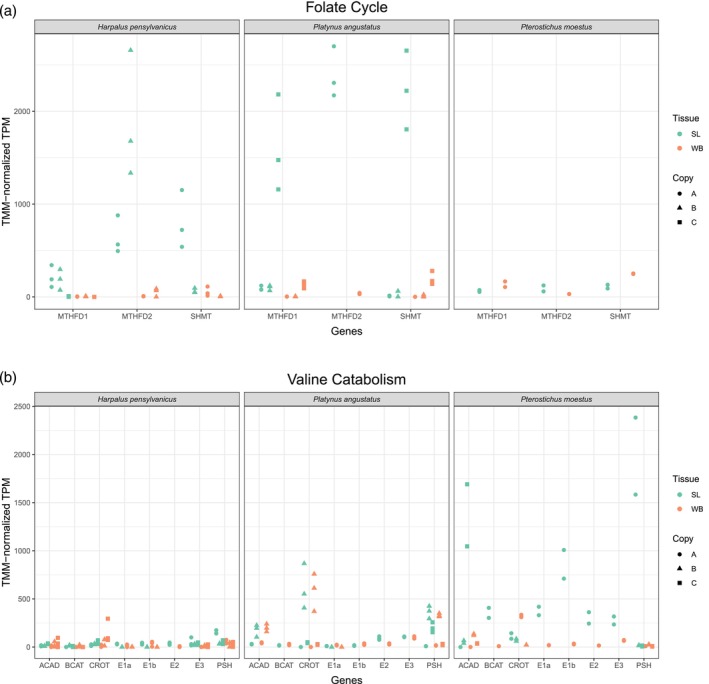
(a, b) Expression of key genes involved in the folate cycle of one‐carbon metabolism (a) and the valine catabolic pathway (b). Genes are listed along the x‐axis whereas the y‐axis measures TMM‐normalised TPM gene expression values. Teal points (left side of the gene bins) represent secretory lobe samples and orange points (right side of the gene bins) represent whole body samples. Although all of these genes are likely single‐copy orthologs, for reasons discussed in this work, de novo assembly annotated multiple copies of most genes, represented here by differently shaped points. Up to the top three most highly expressed gene copies are displayed. This excluded no upregulated genes.

In the secretory lobes of both formic acid producers, *Harpalus pensylvanicus* and *Platynus angustatus*, we found evidence for the upregulation of all three genes involved in the core of the folate cycle of C‐1 metabolism (Figures 3a and 4a). We define upregulation as a significant increase in gene expression in the secretory lobes relative to the rest of the body. Specifically, these genes are serine hydroxymethyltransferase (*SHMT*), bifunctional methylenetetrahydrofolate dehydrogenase/cyclohydrolase (*MTHFD2*) and trifunctional methylenetetrahydrofolate dehydrogenase/cyclohydrolase, formyltetrahydrofolate synthetase (*MTHFD1*) (Brosnan & Brosnan, 2016; Fox & Stover, 2008; Meiser et al., 2018). In the secretory lobe‐upregulated gene sets of both *H. pensylvanicus* and *P. angustatus*, we found a single upregulated copy of *SHMT*. In *H. pensylvanicus*, we found one upregulated copy of *MTHFD2* and two upregulated copies of *MTHFD1*, whereas *P. angustatus* has one upregulated copy each of *MTHFD2* and *MTHFD1*. Other non‐upregulated copies of these genes were also found in *H. pensylvanicus* and *P. angustatus*. It is unlikely that these multiple copies are truly independent genes, but rather are artefacts of de novo transcriptome assembly from heterozygous, pooled conspecifics or of endosymbiont origin (McManus et al., 2018; Rork et al., 2021). Not only are these three genes upregulated in the secretory lobes of both formic acid producers, but they are exclusively so. That is, they are not upregulated in the secretory lobes of *Pterostichus moestus* (Figure 4a).

We also find evidence for the upregulation of one gene involved in the kynurenine pathway of tryptophan catabolism: kynurenine formamidase. Both *H. pensylvanicus* and *P. angustatus* upregulate (*KFA*) in their secretory lobes, two copies in each upregulated gene set (Badawy, 2017; Han et al., 2012; Kanai et al., 2009; Mehler & Knox, 1950) (Figure 4a). No other genes involved in this pathway were upregulated in either species' secretory lobes. As with folate cycle genes, *KFA* upregulation in the secretory lobes is exclusive to the two formic acid producers. Although we find evidence for the upregulation of 1,2‐dihydroxy‐3‐keto‐5‐methylthiopentene dioxygenase of the methionine salvage pathway in *H. pensylvanicus* secretory lobes, we find no such evidence in *P. angustatus*, nor any evidence for the upregulation of other methionine salvage cycle genes within the secretory lobes (Savarse et al., 1985; Sekowska et al., 2019). No other genes with established roles in formic acid biosynthesis, were identified in either species' upregulated gene sets.

Several Gene Ontology terms are enriched exclusively in the secretory lobes of formic acid producers. Amongst those under the ontological category ‘Biological Process’ are ‘Tetrahydrofolate metabolic process’, ‘Folic acid‐containing compound metabolic process’ and ‘One‐carbon metabolic process’, all which map to the core genes of the folate cycle. ‘Carboxylic acid metabolic process’, ‘Organic acid metabolic process’ and ‘Oxoacid metabolic process’ are also exclusively enriched in these species' secretory lobes, all of which again map to genes involved in the folate cycle, as well as the kynurenine pathway. Terms exclusively enriched under the ‘Molecular Function’ category are even more specific, listing all primary functions of MTHFD1 and MTHFD2: ‘Methenyltetrahydrofolate cyclohydrolase activity’, ‘Methylenetetrahydrofolate dehydrogenase (NADP+) activity’ and ‘Formate‐tetrahydrofolate ligase activity’. Although not specific to KFA, the exclusively enriched term ‘Hydrolase activity, acting on carbon–nitrogen (but not peptide) bonds’ does refer to the activity of this enzyme according to the gene IDs mapped to it by GOSeq.

### Candidate genes and pathways involved in methacrylate biosynthesis

In the secretory lobes of *Pterostichus moestus*, we find all but one gene involved in the valine catabolic pathway through the generation of methacrylyl‐CoA upregulated (Figures 3b and 4b) (Lange et al., 2004; Wanders et al., 2010). These are branched‐chain‐amino acid aminotransferase *(BCAT)*, the alpha and beta subunits of the E1 subunit of the branched‐chain alpha‐keto dehydrogenase complex (*E1a* and *E1b*), the E2 subunit of the branched‐chain alpha‐keto dehydrogenase complex (*E2*) and a short‐chain specific acyl‐CoA dehydrogenase (*ACAD*) (Brosnan & Brosnan, 2006; Kochevenko et al., 2012). Only one copy of each gene is upregulated in this species' secretory lobes. These genes are also exclusively upregulated in the secretory lobes of *P. moestus*, our sole methacrylic acid producer. That is, they are not upregulated in the secretory lobes of *H. pensylvanicus* or *P. angustatus*. The one gene in this pathway not significantly upregulated in the secretory lobes of *P. moestus* is the E3 subunit of the branched‐chain alpha‐keto dehydrogenase complex (logFC = 2.44, FDR = 3.8E‐3). Crotonase, which converts methacrylyl‐CoA to 3‐Hydroxyisobutyryl‐CoA, is not significantly upregulated either (logFC = 2.13, FDR = 5.5e‐3).

**FIGURE 5:**
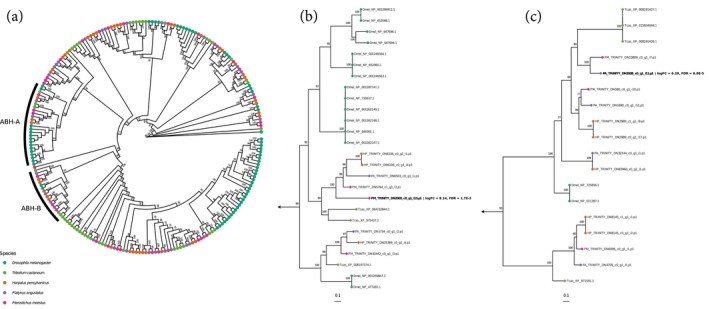
(a, b) Maximum‐likelihood phylogeny of the α/β‐hydrolase (ABH) fold gene family, reconstructed via IQ‐TREE2 from *Tribolium castaneum* (green), *Drosophila melanogaster* (teal), *Harpalus pensylvanicus* (orange), *Platynus angustatus* (purple) and *Pterostichus moestus* (magenta) sequences (a). Ultrafast bootstrap support values (5000 replicates) shown at nodes. Subclades ABH‐A (b) and ABH‐B (c) are annotated in the full phylogeny and shown separately.

To our knowledge, no enzyme has been implicated in the conversion of methacrylyl‐CoA to methacrylic acid in insects. Such an enzyme would have thioesterase activity, capable of cleaving the CoA and acyl moieties to form free CoA and the corresponding acid (Grevengoed et al., 2014). We do find one secretory lobe‐upregulated gene which may have such a function: a serine hydrolase. Serine hydrolases comprise a large family of enzymes, many of which function as acyl‐CoA hydrolases. This upregulated serine hydrolase contains the same α/β‐hydrolase (ABH) domain found in other acyl‐CoA thioesterases, thus making it plausible that this enzyme could hydrolyse methacrylyl‐CoA to methacrylic acid and CoA (Grevengoed et al., 2014). However, due to the lack of high‐quality annotations for the genes in clade ABH‐A of the larger ABH gene family, we cannot make any generalisations on that basis alone (Figure 5a,b). Clade ABH‐B, which is relatively closely related to ABH‐A and contains one upregulated sequence from *P. angustatus*, is likely comprised of lysophosphatidylserine lipases, which have acylglycerol lipase (esterase) and phospholipase (phosphoesterase) activity (Kelkar et al., 2019; Navia‐Paldanius et al., 2012) (Figure 5a,c). They have no known thioesterase activity, however, leaving the function of genes in clade ABH‐A unknown.

Several gene ontology (GO) terms are enriched exclusively in the secretory lobes of *P. moestus*. Under ‘Biological Process’ are the closely related terms ‘branched‐chain amino acid catabolic process’ and ‘branched‐chain amino acid metabolic process’, both of which describe the breakdown of branch‐chain amino acids to their various acyl‐CoA derivatives. Under ‘Cellular Component’ are terms relating specifically to the BCKDH complex, namely ‘dihydrolipoyl dehydrogenase complex’, ‘mitochondrial alpha‐ketoglutarate dehydrogenase complex’, ‘tricarboxylic acid cycle enzyme complexz’ and ‘mitochondrial tricarboxylic acid cycle enzyme complex’. This suggests the enrichment of genes involved in such processes, all of which relate to L‐valine catabolism and methacrylyl‐CoA biosynthesis.

### Candidate genes involved in carboxylic acid transport

As important as the ability to synthesise defensive chemicals is to carabids, so too is their ability to secrete them from their secretory lobes into the lumens of the collecting ducts and reservoirs. In our carboxylic acid producing taxa, this may be accomplished via monocarboxylate symporters, such as those of the sodium:solute symporter family (SSF) (Ganapathy et al., 2008; Jung, 2002; Moschen et al., 2012).

**FIGURE 6:**
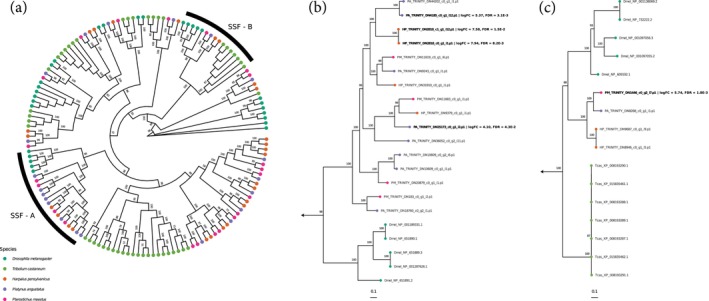
(a, b) Maximum‐likelihood phylogeny of the sodium:solute symporter (SSF) gene family, reconstructed via IQ‐TREE2 from *Tribolium castaneum* (green), *Drosophila melanogaster* (teal), *Harpalus pensylvanicus* (orange), *Platynus angustatus* (purple) and *Pterostichus moestus* (magenta) sequences (a). Ultrafast bootstrap support values (5000 replicates) shown at nodes. Subclades SSF‐A (b) and SSF‐B (c) are annotated in the full phylogeny and are shown separately.

Phylogenetic reconstruction of the sodium:solute symporter gene family suggests that members of two distinct clades may be important for monocarboxylate transport in the secretory lobes of *Harpalus pensylvanicus*, *Platynus angustatus* and *Pterostichus moestus* (Figure 6a–c). Here, we denote these clades as SSF‐A and SSF‐B. Both SSF‐A and SSF‐B contain gene family members from all three species, but the genes upregulated in the secretory lobes of *H. pensylvanicus* and *P. angustatus* are found exclusively in SSF‐A (98% UFBS) whereas the gene upregulated in the secretory lobes of *P. moestus* is exclusive to SSF‐B (100% UFBS). Within SSF‐A, the two upregulated *H. pensylvanicus* genes are sister (100% UFBS) and are themselves sister to two *P. angustatus* genes, one upregulated and one not (100% UFBS). This four‐gene clade is nested within a clade containing a second upregulated *P. angustatus* gene (99% UFBS) (Figure 6a,b). The one upregulated *P. moestus* sequence is upregulated in a distantly related clade (SSF‐B) (Figure 6a,c). The upregulation of putatively orthologous sodium:solute symporters in the two formic acid producers and a relatively distantly related sodium:solute symporter in the methacrylic acid producer suggests that if they are involved in the transport of the primary defensive chemicals into the secretory lobe lumens, they may transport different compounds. However, it is not clear from the *Drosophila* and *Tribolium* genes found in these clades which classes, sizes and polarities of solutes they are likely to transport.

## DISCUSSION

### Defensive‐grade formic acid is likely biosynthesised similarly in *Harpalus* and *Platynus*

The results of our differential gene expression analyses suggest two pathways may be involved in defensive‐grade formic acid biosynthesis in both *Harpalus pensylvanicus* and *Platynus angustatus*. The primary candidate pathway thought to be involved is the folate cycle of one‐carbon metabolism, which has also been implicated in formic acid biosynthesis in ants (Hefetz & Blum, 1978a; Hefetz & Blum, 1978b; Rork et al., 2021). The core of this cycle is comprised of three enzymes: SHMT, MTHFD2 and MTHFD1 (Brosnan & Brosnan, 2016; Fox & Stover, 2008; MacFarlane et al., 2009). L‐serine and tetrahydrofolate are necessary inputs to the pathway, being the one‐carbon donor and the one‐carbon carrier, respectively (Hefetz & Blum, 1978a; Hefetz & Blum, 1978b). The stable intermediates in this pathway, 5,10‐methylenetetrahydrofolate (5,10‐mTHF) and 10‐formyltetrahydrofolate (10‐fTHF), are also intermediates in the biosynthesis of thymidylate and purines respectively (Ben‐Sahra et al., 2016; Carreras & Santi, 1995; Hartman & Buchanan, 1959). When the former is reduced by methylenetetrahydrofolate reductase (MTHFR), the 5‐methyltetrahydrofolate (5‐mTHF) product is also utilised to regenerate methionine from homocysteine via methionine synthase (Zheng et al., 2019). Excess formate is typically exported from the cell into the extracellular space (as in carabid beetles secretory lobes and oxidative cancer cells) or is used in other metabolic processes (Meiser et al., 2016).

The second candidate pathway potentially involved in defensive‐grade formic acid biosynthesis is the kynurenine pathway. This pathway begins by the oxidation of the imidazole ring of tryptophan, generating N‐formylkynurenine (Badawy, 2017; Han et al., 2012; Mehler & Knox, 1950). N‐formylkynurenine is then hydrolysed by kynurenine formamidase, cleaving off the formyl group as formate and generating L‐kynurenine. L‐kynurenine can then be used to synthesise a variety of compounds, most notably nicotinamide adenine dinucleotide (NAD) (Badawy, 2017).

Of these two, we view it more likely that the folate cycle is the primary pathway responsible for defensive‐grade formic acid biosynthesis. L‐serine is less bioenergetically expensive than L‐tryptophan, making it a more suitable precursor for the biosynthesis of relatively large quantities of formate (Barik, 2020; Moura et al., 2013; Wu et al., 2020). L‐tryptophan is both an essential amino acid and is amongst the least abundant in organisms (Moura et al., 2013). L‐serine, on the other hand, is comparatively more abundant and can be biosynthesised by animals from L‐glycine or 3‐phosphoglycerate (Moura et al., 2013; Wu et al., 2020). Also, by virtue of being a cycle, the only outputs of the folate cycle when it is primarily involved in biosynthesising formate are formate, tetrahydrofolate, L‐glycine, NAD(P)H and ATP (Fox & Stover, 2008). Formate is exported, tetrahydrofolate can be reused by the folate cycle and NAD(P)H and ATP are likely needed in abundance due to the high metabolic activity of the glands. On the other hand, the kynurenine pathway is acyclic and would produce comparatively specific metabolites that would likely need to serve a niche metabolic purpose or be degraded (Badawy, 2017). Given the above, we view this pathway as the less probable of the two options. The involvement of either pathway should be viewed as untested hypotheses given the lack of functional validation data to establish causality.

Previous work suggested the methionine salvage cycle as a potential albeit unlikely pathway for formate biosynthesis in *H. pensylvanicus*, due to its upregulation of a formate‐generating 1,2‐dihydroxy‐3‐keto‐5‐methylthiopentene dioxygenase in the secretory lobes (Rork et al., 2021). While this gene is still upregulated in our new *H. pensylvanicus* assembly, it is not in *P. angustatus*. We consider it improbable that the methionine salvage cycle has any significant involvement in formate biosynthesis given its complexity and the lack of gene expression data in support of it.

Importantly, while *H. pensylvanicus* and *P. angustatus*, members of the same subfamily, seem to upregulate the same genes for formic acid biosynthesis in their secretory lobes, they likely did not converge upon this phenotype. Prior work suggests that the formic acid chemotype was derived from a common ancestor in these two genera (Ober & Heider, 2010; Ober & Maddison, 2008; Will et al., 2000). However, our data can be seen as two independent data points supporting the hypothesis that the folate cycle and perhaps the kynurenine pathway are involved in defensive‐grade formic acid biosynthesis in some ground beetle taxa.

### Valine catabolism may underlie methacrylic acid biosynthesis in *Pterostichus*

The results of our differential gene expression analyses for *Pterostichus moestus* suggest the valine catabolic pathway may be involved in the biosynthesis of defensive‐grade methacrylic acid. This pathway begins with the transamination of L‐valine to form 2‐oxoisovaleric acid, which is then decarboxylated and covalently bound to coenzyme A to form isobutyryl‐CoA (Brosnan & Brosnan, 2006; Wanders et al., 2010). Isobutyryl‐CoA is then reduced to methacrylyl‐CoA, which can be further metabolised to ultimately form succinyl‐CoA, an intermediate in the citric acid cycle (Wanders et al., 2010). Necessarily, an enzyme capable of hydrolysing the thioester bond of methacrylyl‐CoA would instead be capable of generating the final methacrylic acid product detected in the pygidial glands of *P. moestus*. We hypothesise that this enzyme is a serine hydrolase, some of which have been functionally characterised as having acyl‐CoA ligase or thioesterase activity (Grevengoed et al., 2014; Long & Cravatt, 2011). The probable serine hydrolase upregulated in the secretory lobes of *P. moestus* specifically belongs to the ABH family which does contain members having thioesterase activity, possibly capable of hydrolysing methacrylyl‐CoA to methacrylic acid (Long & Cravatt, 2011) (Figure 5a–c). As above, this of course remains speculative and would require functional characterisation to confirm.

Interestingly, several other compounds present in the pygidial secretions of *P. moestus* are likely generated through the wider catabolism of branched‐chain amino acids. L‐valine, L‐leucine and L‐isoleucine can all be reduced to isobutyryl‐CoA, isovaleryl‐CoA and (S)‐2‐methylbutanoyl‐CoA, respectively, via BCAT and the BCKDH complex (Brosnan & Brosnan, 2006; Kochevenko et al., 2012; Wanders et al., 2010). Generalist short‐chain acyl‐CoA dehydrogenases or substrate‐specific dehydrogenases may further reduced these compounds to methacrylyl‐CoA, 3‐methylcrotonyl‐CoA and tiglyl‐CoA, respectively. Tiglyl‐CoA is further converted to Propanoyl‐CoA. Isobutyryl‐CoA, methacrylyl‐CoA, tiglyl‐CoA and propanoyl‐CoA are all acyl‐CoA esters of isobutyric acid, methacrylic acid, tiglic acid and propanoic acid, respectively (Figure 2b,c). Throughout the branched‐chain carboxylic acid‐defended Carabidae, several other carboxylic acids, such as crotonic acid, isovaleric acid and angelic acid, are likely also produced via these interconnected pathways (Kanehisa & Murase, 1977; Moore & Wallbank, 1968). The relationship between these pathways and the defensive chemistry of such Carabidae is further supported by isotope incorporations data. The stable isotope D8‐L‐valine is incorporated into methacrylic and isobutyric acids in *Scarites subterraneus*; L‐[2,3,4,4‐(2)H (4)]‐isoleucine is incorporated into tiglic, 2‐methylbutyric and ethacrylic acids in *Pterostichus californicus*; and L‐[U‐14C]‐valine, L‐[U‐14C]‐leucine and L‐[U‐14C]‐isoleucine were incorporated into methacrylic acid, 3‐methylcrotonic acid and tiglic acid respectively in *Carabus yaconinus* (Adachi et al., 1985; Attygalle et al., 1991; Attygalle et al., 2006).

### Formic and methacrylic acid producers may export defensive chemicals via sodium:solute symporters

Although it is uncertain which genes are involved in defensive chemical export from the secretory lobes in all taxa, we assess the most likely candidates to belong to the sodium:solute symporter family (SSF). This family includes proteins involved in monocarboxylate transport (Ganapathy et al., 2008; Jung, 2002; Moschen et al., 2012). It is noteworthy that the two formic acid producers, *H. pensylvanicus* and *P. angustatus*, upregulate closely related (possible orthologous) sodium:solute symporter homologues (Figure 6a,b) while the methacrylic acid producer, *P. moestus*, upregulates a comparatively distantly related sodium:solute symporter homologue (Figure 6a,c). Alternatives to SSF transporters include a variety of organic anion and cation transporters, many of which are members of the functionally diverse major facilitator superfamily (MFS) (Quistgaard et al., 2016). However, those few upregulated in the secretory lobes of *Platynus angustatus* generally seem unlikely to be involved in formate transport, but rather trehalose transport. Thus, while we view SSF transporters as the most likely to be involved in defensive chemical export, other gene family members may play important roles as well.

### Future work is necessary to fully characterise the biosynthetic pathways involved in defensive chemical production

The candidate genes and biosynthetic pathways identified through our comparative transcriptomics study align with known mechanisms of defensive formic acid biosynthesis in ants and methacrylic acid biosynthesis in carabid beetles (Adachi et al., 1985; Attygalle et al., 1991; Hefetz & Blum, 1978a). However, we propose that functional assays, to validate the relationship between these candidate genes and defensive chemical biosynthesis, are conducted in the future.

Isotopic precursor incorporation has long been used as a mechanism for validating the role of precursors and pathways in the biosynthesis of metabolites, especially in insects, including carabid beetles (Adachi et al., 1985; Attygalle et al., 1991; Attygalle et al., 2020). The generation of a labelled metabolite following the incorporation of such an isotopically labelled precursor may be interpreted as causal evidence for the role of said precursor in that metabolite's biosynthesis. We propose that L‐serine‐^13^C_3_ would be ideal for validating the role of the folate cycle in formic acid biosynthesis, as it is the β‐carbon of L‐serine and one of the two covalently bound hydrogens incorporated into formic acid (Meiser et al., 2016). To test the involvement of the kynurenine pathway, L‐tryptophan‐^13^C_2_, would be suitable as it is C_2_ of the imidazole ring which is incorporated into formic acid (Han et al., 2012). In the methacrylic acid producing taxa, L‐valine‐d_8_ or L‐valine‐^13^C_5_ are plausible labelled precursors, as a significant portion of L‐valine is incorporated into methacrylic acid via L‐valine degradation (Attygalle et al., 1991).

We do not view gene knockout as a viable strategy for functional validation in these systems, as all candidate genes and pathways play crucial roles in primary metabolism. The folate cycle and kynurenine pathways are involved in purine and NAD^+^ biosynthesis respectively, whereas L‐valine degradation is critical to the biosynthesis of succinyl‐CoA, amongst other important biological processes (Ben‐Sahra et al., 2016; Wanders et al., 2010). Thus, it is likely that knockouts would be debilitating if not lethal to embryos. Gene knockdown by RNAi, however, may be viable as its effects would be relatively acute in adults. To test the involvement of the folate cycle and kynurenine pathways, genes of interest include MTHFD1 and KFA, respectively, given their roles in the formic acid‐generating steps of these pathways. Given that some domains present in MTHFD1 are also found in MTHFD2, it is plausible that dsRNA targeting the former would also affect the latter (Rork et al., 2024), a confounding off‐target effect (Jackson et al., 2003; Kulkarni et al., 2006). For methacrylic acid biosynthesis via L‐valine degradation, ACAD, responsible for generating methacrylyl‐CoA, is a reasonable choice. RNAi studies targeting L‐valine degradation should also target genes with potential involvement in the crucial step of methacrylic acid biosynthesis from methacrylyl‐CoA, such as the probable serine hydrolases, mentioned herein. Again, off‐target effects and other confounding factors would need to be considered throughout the experiment to ensure any acidless phenotypes observed via GC–MS are due to the knockdown of target genes and not due to general metabolic disruption.

In addition to gene knockdown, enzyme inhibition would be an interesting albeit less common mechanism for testing the role of these pathways in defensive chemical biosynthesis. Compounds such as methotrexate are widely used in chemotherapy regimens to inhibit the folate cycle and, at least in theory, could be injected or fed to beetles to knock down not gene expression, but enzyme and pathway activity (Meiser et al., 2018). Of course, like RNAi, the confounding effects of injecting or feeding a compound like methotrexate to carabids would need to be carefully considered given the novelty of such a use.

## EXPERIMENTAL PROCEDURES

### Beetle collection

*Platynus angustatus* and *Pterostichus moestus* specimens were collected at Stone Valley Recreation Area in Huntingdon County, Pennsylvania throughout the summer and fall of 2019. Beetles were kept in plastic containers with coconut fibre substrate, were fed a diet of pecans and dog food (Castor & Pollux Organic Cookies, Chicken Recipe) and were provided with wet paper towels for water. Both species, especially *P. moestus*, were observed to be extraordinarily cannibalistic even when fed and were thus isolated into individual containers.

### GC–MS analysis of pygidial gland contents

Gland exudates were collected for *Pterostichus moestus* and *Platynus angustatus* as described in previous work (Rork et al., 2020). Briefly, for each species, the hind legs of two beetles were pinched, causing them to spray into 2 mL autosampler vials containing anhydrous dichloromethane (Sigma‐Aldrich). While we were able to confidently determine the sexes of *Pterostichus moestus* prior to collecting their gland exudates and thus collect sex‐specific sprays, we were unable to do so for *Platynus angustatus*. A typical external character used to determine sex in Carabidae is the size of the foretarsi, males often having broader foretarsi than females, but this dimorphism was vague‐to‐absent in *P. angustatus*. making reliable identification difficult without a reference or dissection. Gland extracts were analysed on a 30 m × 0.25 mm × 0.25 μm ZB‐WAX column installed in a Shimadzu 17A gas chromatograph coupled to a QP5050 mass spectrometer. The oven temperature was held at 40°C for 4 minutes and increased at 10°C/min to a final temperature of 240°C and held for 5 minutes.

### Pygidial gland dissections

*Platynus angustatus* and *Pterostichus moestus* specimens were dissected as described in previous work (Rork et al., 2021). Briefly, hind legs of beetles were pinched to induce a defensive spray response. After waiting 30 min, beetles were separated at the prothoracic‐mesothoracic junction and were stored in RNAlater Stabilization Solution (Invitrogen) at −80°C until dissection. Specimens were dissected under an Olympus SZX16 stereomicroscope using fine‐tipped forceps in watch glasses filled with RNAlater Stabilization Solution. All instruments, surfaces and tools were cleaned with 70% ethanol (Kopec) and RNase Away Decontamination Reagent (Thermo Scientific) prior to dissection. Forceps were also flamed prior to RNase Away treatment. Beetles were dissected and pooled into two tissue types: secretory lobe tissue and whole‐body tissue without secretory lobes. Wings and elytra were stored separately as vouchers. Three biological replicates were collected for both taxa.

### RNA extractions, library preparation and Illumina sequencing

*Platynus angustatus* and *Pterostichus moestus* samples were processed as described in previous work (Rork et al., 2021). Briefly, total RNA was extracted from both secretory lobe samples and whole‐body samples using a standard TRIzol protocol and isolated using a DirectZol RNA MiniPrep Kit. PolyA‐selected, strand‐specific libraries were prepared using an Illumina Nextera Library Preparation kit. *P. angustatus* and *P. moestus* libraries were sequenced on an Illumina NovaSeq 6000 (two lanes, PE, 150 bp) at target reads depths of 25‐30 M/library.

### Computing resources and bioinformatics software

All major bioinformatic analyses conducted as part of this study were carried out through the ROAR COLLAB (RHEL 7/8) at The Pennsylvania State University unless otherwise noted. Aside from certain R packages and their dependencies mentioned herein, all bioinformatics software was installed via the miniconda/anaconda package managers. A full list of software, packages, versions and conda builds used in this work, as well as associated scripts and data files, can be found in SupplementaryData.

### Quality assessment of reads and trimming

The *Harpalus pensylvanicus* RNA‐Seq data generated in previous work (see Rork et al., 2021) was reanalysed as part of this study. Prior to quality assessment, FASTQ files generated from libraries split across lanes (i.e. *Platynus angustatus* and *Pterostichus moestus*) were concatenated sample‐wise. For all species, initial FASTQC (v0.11.9) reports were generated for each sample and were inspected for data quality issues. After quality assessment, adapter content was examined using BBTools' (v38.90) bbduk.sh script using the adapters.fa file as a reference database (Bushnell, 2014). Adapters were then trimmed using bbduk.sh using the literal adapter strings as references. The 3′ ends of reads were then trimmed to Q10. Poly‐G tails were also trimmed and reads containing ambiguous bases (Ns) were discarded, as were all reads shorter than 75 bp post‐trimming. FASTQC reports were subsequently generated for each trimmed library to ensure detectable adapters, N‐containing reads and short reads were adequately removed without any trimming‐induced degradation in library quality.

### Transcriptome assembly and quality assessment

Transcriptomes were assembled de novo using Trinity RNA‐Seq (v2.12) (Grabherr et al., 2011; Haas et al., 2013). Default settings were used for each assembly with two notable exceptions: read orientation (−‐SS_lib_type) was specified as ‘RF’ due to the strand‐specific nature of our libraries and minimum contig length (−‐min_contig_length) was set to 300 bp.

ExN50 values and transcript length statistics were generated via the ‘contig_ExN50_statistic.pl’ and ‘TrinityStats.pl’ scripts respectively. The PtR script was used to assess replicate‐wise and sample‐wise correlations within species. BUSCO was run on each transcriptome using default settings aside from the analysis mode being set to ‘transcriptome’ (Simāo et al., 2015). The insect_odb9 database was used as the reference set of benchmark universal single‐copy orthologs.

### Coding sequence prediction and functional annotation

Coding sequences were predicted ab initio from transcripts and were in silico translated to their respective proteins via TransDecoder (v5.5.0) (Haas et al., 2013). To determine homology of assembled transcripts and their predicted proteins BLASTX and BLASTP (v2.11.0) searches were run against the UniProtKB database using the full set of assembled transcripts and their predicted proteins as queries, respectively (Altschul et al., 1990). HMMScan (v3.3.2) searches were also run against the Pfam‐A protein database using the full set of predicted proteins to assess protein domain content (Eddy, 1998; Finn et al., 2011; Punta et al., 2012). For all BLAST and HMMScan searches, parameters were left as defaults except the e‐value threshold set to 1e‐10. Both the UniProtKB and Pfam databases were originally downloaded in March 2021 from the European Bioinformatics Institute's FTP site. At the time of analysis, Trinotate was unable to access certain online datasets (Haas et al., 2013). For this reason, a custom script was created to generate analogous databases. Trinotate (v3.2.2) databases were later generated for each species post‐update.

### Read quantification, differential gene expression analyses and GO enrichment

Transcript quantification was carried out using the Kallisto (v0.46.2) pseudoalignment‐based strategy via the ‘align_and_estimate_abundance.pl’ script included as part of Trinity RNA‐Seq's downstream analysis utilities (Bray et al., 2016). All settings were left as default. Transcripts and gene expression matrices were subsequently generated using the ‘abundance_estimates_to_matrix.pl’ script, all settings again left as default.

Kallisto quantification results were used as inputs to the ‘run_DE_analysis.pl’ script, used here to conduct differential gene expression analyses between tissues of each species (Bray et al., 2016). Specifically, contrasts were performed between secretory lobe (SL) samples and whole body (WB) samples in all species to identify genes significantly upregulated in the former relative to the latter. Voom was used to log2‐transform gene‐wise count data to log2‐counts per million, and limma (v3.42.0) was used to identify differentially expressed genes from these data (Law et al., 2014; Ritchie et al., 2015). Genes were considered differentially upregulated in the secretory lobes at logFC >4.0 and FDR <0.05 (Benjamini & Hochberg, 1995; Storey, 2002). This logFC cut‐off, high compared to the usual cutoff of 1.5–2.0, was chosen to focus on the most upregulated genes in the secretory lobes. Notable genes logFC values <4.0 are also discussed where appropriate.

GO enrichment analyses were run using GOSeq (v1.38.0) via the run_goseq.pl. script, which was modified such that all GO Terms would be printed rather than only those with FDR <0.05 (Benjamini & Hochberg, 1995; The Gene Ontology Consortium, 2000; Young et al., 2010). Lists of enriched and depleted GO terms for the secretory lobes relative to the whole bodies were generated for each species. The go_basics.obo file was downloaded manually, as the GO Consortium link changed between Trinity version releases (Aug. 2021). Terms were classified as significantly enriched at FDR <0.05. GO enrichment results were summarised using the R (v4.1.3) package RRVGO (v1.4.4) (Sayols, 2020).

### Identification of chemotype‐specific enriched GO terms

To better assess which processes are important for chemical defence in a specific chemotype, we conducted multispecies combinatorial analyses to identify those GO terms commonly enriched in the secretory lobes of certain taxa. Specifically, we identified enriched GO terms exclusive to the secretory lobes of our formic acid producers and enriched GO terms exclusive to the secretory lobes of our methacrylic acid producer. This was done by piping lists of enriched GO terms of each species into successive grep or inverse grep commands, at each step filtering out terms found in the non‐target chemotype, thus leaving only terms shared by members of a chemotype at the end of the filtering process. To generate our list of formic acid‐specific GO terms, for example, we first file grepped the list of secretory lobe‐enriched GO IDs of *Harpalus pensylvanicus* against the list of secretory lobe‐enriched GO IDs of *Platynus angustatus*. This gave us a subset of GO Terms enriched in both species' secretory lobes. We then inverse grepped this list against the secretory lobe‐enriched GO IDs of *P. moestus*, effectively removing from the initial list any secretory lobe‐enriched GO IDs shared between ‘*H. pensylvanicus* AND *P. angustatus*’ and *P. moestus*. This final list of GO IDs thus represented formic acid producer‐specific secretory lobe‐enriched GO IDs, which were then matched back to their respective GO Terms for interpretation. The same approach was used for *P. moestus*, only that inverse grep was exclusively used since we had no secondary methacrylic acid producer.

### Phylogenetic reconstruction of SSF and ABH gene families

Understanding the phylogenetic relationships between upregulated genes is necessary to assess the patterns underlying their evolution, their co‐option and their putative functions. Profile HMMs (pHMM) were downloaded for the sodium:solute symporter protein family (PF00474) and the alpha/beta hydrolase family (PF00561) from InterPro. The full set of coding and protein sequences from *Drosophila melanogaster* (GCF_000001215.4) and *Tribolium castaneum* (GCF_000002335.3) were downloaded from the RefSeq FTP server and the sequence headers shortened to species abbreviations and accessions. The longest coding and protein sequence isoforms per gene of all three carabids, *D. melanogaster* and *T. castaneum* were then concatenated into one multifasta file per sequence type.

Each pHMM was searched against the concatenated protein multifasta file using hmmsearch (HMMER v3.1b2) with an e‐value reporting threshold of 1E‐10. Accessions of hits were used to extract corresponding sequences from the concatenated coding and protein multifasta files (as coding and protein sequence accessions are identical in NCBI). Extracted coding sequences were aligned with MAFFT (v7.505) with the ‐maxiterate 1000 and ‐localpair parameters set, alignments were trimmed with trimAl (v1.4.rev15) with the ‐automated1 parameter set and gap‐rich sequences (comprised of >90% gap) were removed (Capella‐Gutiérrez et al., 2009; Katoh & Standley, 2013).

Maximum‐likelihood phylogenies were constructed for both gene families using IQ‐Tree2 (v2.1.4‐beta) with the following parameters set: ‐m MFP (standard model selection followed by tree inference) ‐B 5000 (5000 ultrafast bootstrap replicates) ‐merit BIC (BIC criterion used for model selection) (Minh et al., 2020). Ten independent runs were carried out per gene family (−‐runs 10) and the topology with the highest likelihood was chosen. Maximum‐likelihood tree files were imported to R (v4.1.3) and the ggplot2 (v3.4.2) and ggtree (v3.2.1) packages, principally, were used to annotate the phylogenies (Paradis et al., 2004; Yu et al., 2017).

## CONCLUSIONS

The Carabidae are a taxonomically and biochemically diverse lineage whose chemical defence strategies have fascinated evolutionary biologists since the time of Darwin (Darwin, 1846). Over the past several decades, considerable efforts have been undertaken to characterise the diversity of these defensive chemicals, leading to the accrual of hundreds of known compounds from carboxylic acids to phenolics, from sulphides to cyanide and more. While there has been much interest in how ground beetles (and insects more broadly) synthesise their defensive compounds, few pathways have been deduced and only for a handful of taxa. Here, we provide transcriptomic evidence suggesting likely pathways for the biosynthesis of the two most common defensive chemicals across the Carabidae, formic acid and methacrylic acid. This work builds upon prior research suggesting that defensive‐grade formic acid is biosynthesised by insects from serine via the folate cycle and that defensive‐grade methacrylic acid is biosynthesised in Carabidae through the catabolism of valine. We also provide evidence for a pathway not previously suggested to play a major role in formic acid biosynthesis in insects, the kynurenine pathway.

## AUTHOR CONTRIBUTIONS

**Adam M. Rork:** Conceptualization; investigation; funding acquisition; writing – original draft; writing – review and editing; visualization; methodology; software; formal analysis; data curation. **Sihang Xu:** Investigation; writing – original draft; methodology; visualization; writing – review and editing; formal analysis; conceptualization; data curation. **Athula Attygalle:** Writing – original draft; writing – review and editing; methodology; project administration; supervision; resources; funding acquisition; conceptualization; visualization. **Tanya Renner:** Writing – original draft; funding acquisition; methodology; writing – review and editing; project administration; supervision; resources; visualization; conceptualization.

## ETHICS STATEMENT

As all beetles were collected on property of The Pennsylvania State University, permits were not needed. None of the authors declare any conflicts of interest.

## Supporting information


**Table S1.** Summary statistics on raw sequencing data output and data retention post‐trimming for each species.
**Table S2.** Summary statistics on de novo transcriptome assembly size, transcript contiguity and gene content for each species.
**Table S3.** Summary statistics on BUSCO completeness for each species.
**Table S4.** Summary statistics on coding sequence prediction and functional annotation for each species. BLAST searches were run against the UniProtKB database whereas HMMScan searches were run against the Pfam‐A database.
**Table S5.** Summary statistics on the pseudoalignment rates of reads to their respective transcriptome assemblies and differential gene expression analyses for each species.

## Data Availability

All supplementary data, scripts and supplementary tables are available through Penn State Scholar Sphere: https://scholarsphere.psu.edu/resources/bf571e7d-95f7-40b3-b507-69ed5b971e97.
