# Supplementary material for: The molecular mechanisms of defensive‐grade organic acid biosynthesis in ground beetles
Source: Insect Mol Biol. 2025 Feb 10;34(5):593–607. doi: 10.1111/imb.12984 (PMC12419134; doi:10.1111/imb.12984)
Supplement: Supplementary file 1 — Table S1. Summary statistics on raw sequencing data output and data retention post‐trimming for each species. Table S2. Summary statistics on de novo transcriptome assembly size, transcript contiguity and gene content for each species. Table S3. Summary statistics on BUSCO completeness for each species. Table S4. Summary statistics on coding sequence prediction and functional annotation for each species. BLAST searches were run against the UniProtKB database whereas HMMScan searches were run against the Pfam‐A database. Table S5. Summary statistics on the pseudoalignment rates of reads to their respective transcriptome assemblies and differential gene expression analyses for each species. [file IMB-34-593-s001.docx]

**SUPPLEMENTARY TABLES**

|  | *H. pensylvanicus* | *P. angustatus* | *P. moestus* |
| --- | --- | --- | --- |
| Reads (pre-trim) | 3.00E+08 | 5.49E+08 | 4.24E+08 |
| Reads out (post-trim) | 2.93E+08 | 5.38E+08 | 4.09E+08 |
| % reads retained | 97.8% | 98.1% | 96.4% |
| Bases in (pre-trim) | 4.53E+10 | 8.26E+10 | 6.39E+10 |
| Bases out (post-trim) | 4.27E+10 | 7.17E+10 | 5.43E+10 |
| % bases retained | 94.3% | 86.8% | 84.9% |

**STable 1.** Summary statistics on raw sequencing data output and data retention post-trimming for each species.

|  | *H. pensylvanicus* | *P. angustatus* | *P. moestus* |
| --- | --- | --- | --- |
| Genes | 93,716 | 63,405 | 49,008 |
| Transcripts | 163,238 | 145,296 | 105,544 |
| Transcript N50 | 1,621 bp | 1,613 bp | 1,837 bp |
| Transcript E90N50 | 1,750 bp | 1,649 bp | 1,682 bp |
| GC Content | 36.9% | 36.3% | 37.3% |
| Assembly Size | 1.67E+08 bp | 1.57E+08 bp | 1.23E+08 bp |

**STable 2.** Summary statistics on *de novo* transcriptome assembly size, transcript contiguity, and gene content for each species.

|  | *H. pensylvanicus* | *P. angustatus* | *P. moestus* |
| --- | --- | --- | --- |
| BUSCO Complete | 97.4% | 98.5% | 98% |
| BUSCO Single | 4.6% | 12.3% | 33.9% |
| BUSCO Duplicated | 92.8% | 86.2% | 64.1% |
| BUSCO Fragmented | 0.9% | 0.6% | 0.9% |
| BUSCO Missing | 1.7% | 0.9% | 1.1% |

**STable 3.** Summary statistics on BUSCO completeness for each species.

|  | *H. pensylvanicus* | *P. angustatus* | *P. moestus* |
| --- | --- | --- | --- |
| Transcripts w/ CDS | 75,652 | 65,054 | 50,311 |
| Coding Sequences | 86,399 | 69,569 | 55,837 |
| Transcripts w/ BlastX Hit | 57,444 | 47,638 | 37,740 |
| Proteins w/ BlastP Hit | 57,106 | 44,472 | 36,654 |
| Proteins w/ HMMScan Hit | 52,161 | 41,949 | 33,362 |

**STable 4.** Summary statistics on coding sequence prediction and functional annotation for each species. BLAST searches were run against the UniProtKB database whereas HMMScan searches were run against the Pfam-A database.

|  | *H. pensylvanicus* | *P. angustatus* | *P. moestus* |
| --- | --- | --- | --- |
| Avg. % pseudoalignment | 68.0% | 75.8% | 81.9% |
| DE genes | 1,479 | 1,198 | 4,358 |
| Genes SLUP | 1,010 | 469 | 488 |

**STable 5.** Summary statistics on the pseudoalignment rates of reads to their respective transcriptome assemblies and differential gene expression analyses for each species.
